# 

**Published:** 1860-10

**Authors:** 


☞ The Chair of Anatomy in the Medical College of the State of South Carolina, so long and ably filled by Professor John E. Holbrook, has been vacated. Dr. Francis T. Miles, for many years Demonstrator of Anatomy in that Institution, fills the Chair thus made vacant.

The Faculty have supplied the place of Dr. Miles, by the appointment of Dr. Samuel Logan, formerly Assistant Demonstrator, to the post of Demonstrator in Chief.

☞ A Committee from the French Academy, to whom was referred in 1859, the consideration of a mixture of Coal-tar and Plaster-of-Paris as a disinfectant application to ulcers, &c., recently reported to the Academy, strongly favoring the use of the mixture in powder, and in the form of poultice, made by combining it with oil. The form of poultice was found most useful in open suppurating abscesses—that of powder to indolent, ill-conditioned ulcers. The mixture, it will be remembered, consists of 100 parts of plaster to one of coal-tar. We think, however, inasmuch as the disinfectant property of the mixture depends, to a large extent, upon the coal-tar, that it might with propriety be combined in larger proportion.

### TO CORRESPONDENTS.

By a recent change in the management of the Journal, its whole business affairs will hereafter come under the supervision of the Editor, and it is therefore requested that ALL Communications, whether relating to the Editorial, Subscription, Advertising or Financial Departments, should be addressed to J. G. WESTMORELAND, or Editor of Medical and Surgical Journal, Atlanta, Ga.

